# Overlapping *Toxoplasma gondii* Genotypes Circulating in Domestic Animals and Humans in Southeastern Brazil

**DOI:** 10.1371/journal.pone.0090237

**Published:** 2014-02-27

**Authors:** Letícia A. Silva, Renata O. Andrade, Ana Carolina A. V. Carneiro, Ricardo W. A. Vitor

**Affiliations:** Department of Parasitology, Institute of Biological Sciences, Universidade Federal de Minas Gerais, Belo Horizonte, Minas Gerais, Brazil; University of Wisconsin Medical School, United States of America

## Abstract

Although several *Toxoplasma gondii* genotyping studies have been performed in Brazil, studies of isolates from animals in the state of Minas Gerais are rare. The objective of this study was to conduct a genotypic characterization of *T. gondii* isolates obtained from dogs, free-range chickens, and humans in Minas Gerais and to verify whether the *T. gondii* genotypes circulating in domestic animals correspond to the genotypes detected in humans. Genetic variability was assessed by restricted fragment length polymorphism at 11 loci (SAG1, 5′+3′SAG2, SAG2 alt, SAG3, BTUB, GRA6, c22-8, c29-2, L358, PK1, and Apico). Twelve different genotypes were identified among the 24 isolates studied, including 8 previously identified genotypes and 4 new genotypes. The genetic relationship of the 24 *T. gondii* isolates, together with the genotypes previously described from 24 human newborns with congenital toxoplasmosis, revealed a high degree of similarity among the genotypes circulating in humans and animals in Minas Gerais. The most common genotypes among these species were BrII, BrIII, ToxoDB #108, and ToxoDB #206. Restricted fragment length polymorphism at the CS3 locus of these 48 isolates showed that the majority of isolates presented alleles I (50%) or II (27%). Isolates harboring allele III at the CS3 locus presented low virulence for mice, whereas those harboring alleles I or II presented higher virulence. These results confirm the utility of marker CS3 for predicting the virulence of Brazilian isolates of *T. gondii* in mice. No association was found between the allele type and clinical manifestations of human congenital toxoplasmosis. This is the first report of *T. gondii* genotyping that verifies the overlapping genotypes of *T. gondii* from humans and animals in the same geographic region of Brazil. Our results suggest that there is a common source of infection to the species studied, most likely oocysts contaminating the environment.

## Introduction

*Toxoplasma gondii* is an obligate, intracellular protozoan parasite distributed worldwide that can infect all warm-blooded animals. Infection in humans is usually asymptomatic, but it can manifest itself in a severe form in cases of congenital toxoplasmosis and in immune-compromised individuals [1].

*T. gondii* isolates obtained from humans and animals from Europe and North America have been classified into one of three genetic clonal lineages, Types I, II, and III [2]. However, studies using multi-locus markers showed high genetic diversity in South American strains, which is absent in North American and European strains [3], [4]. Analysis of the isolates from domestic animals revealed four genotypes considered common clonal lineages in Brazil, BrI BrII, BrIII, and BrIV [5]. Several genotyping studies have been performed on Brazilian isolates of *T. gondii*. However, the majority of isolates are obtained from animal infection [1], and studies of *T. gondii* obtained from humans are rare [6], [7]. Recently, a study conducted in the state of Minas Gerais (Southeastern Brazil) using isolates obtained from newborns revealed a great genetic diversity and the occurrence of Brazilian clonal genotypes [7]. However, genotyping studies of *T. gondii* obtained from domestic animals using the 11 previously proposed markers [8] remain rare in Minas Gerais. Genotyping studies are important for understanding the population structure and parasite phylogeny [8]. In addition, the association between *T. gondii* genotypes obtained from humans and animals in the same region can provide information regarding the epidemiology of this zoonosis and may help identify the sources of infection or means of transmission to humans.

A CS3 marker, located on *T. gondii* chromosome VIIa, was previously shown to be linked to the acute virulence of clonal type I *T. gondii* strains [9]. Moreover, a study of Brazilian *T. gondii* isolates showed that the alleles type I and II at the CS3 locus are strongly linked to mouse virulence of the parasite [5].

In this study, we genetically characterized *T. gondii* isolates obtained from animals and humans in Minas Gerais, and verified whether the genotypes circulating in the animals correspond to those detected in human cases of congenital toxoplasmosis. We also determined whether the allele type at the CS3 locus is associated with the mouse virulence of these isolates. We found overlapping *T. gondii* genotypes circulating in domestic animals and humans, and verified that the alleles I and II at the CS3 marker are associated with higher mouse virulence of the isolates from Minas Gerais.

## Materials and Methods

### Ethics statement

The protocols conducted in this study were approved by the local Animal Ethics Committee (CETEA-Federal University of Minas Gerais, protocols 128/2010).

### Genotypic characterization of *T. gondii* obtained from animals and humans in Minas Gerais

#### DNA from *T. gondii* isolates

DNA from 24 *T. gondii* isolates was used for genotypic characterization by restricted fragment length polymorphism (PCR-RFLP). These samples were previously obtained from studies performed in our laboratory, and originate from the Metropolitan Region of Belo Horizonte (MRBH), Minas Gerais (MG). Twenty samples are from infected animals [10] and four samples are from pregnant women with toxoplasmosis [3]. *T. gondii* isolates D1 to D8 were obtained from the diaphragms of dogs that were naturally infected with leishmaniasis, and euthanized at the Center of Zoonosis Control in Belo Horizonte, MG, from February to June, 2000. Isolates CH1 to CH12 were obtained from the hearts of free-range chickens raised for human consumption in the MRBH residential area, from 2000 to 2003 [10]. SAF, EFP, EGS and RAR isolates were obtained from the amniotic fluid of human cases of congenital toxoplasmosis at the UFMG Hospital das Clínicas, from 1997 to 1999 [3], [11], [12]. The DNA of the 24 isolates was extracted from the tachyzoites recovered from the peritoneum of Swiss mice previously infected with *T. gondii* cysts [3], [10]. To perform this study, DNA samples were re-hydrated with 30 µl ultra-pure water and frozen at 4°C. When a new extraction was necessary, fresh tachyzoites were subjected to DNA extraction with the Wizard Genomic DNA Purification kit (Promega Corporation, Madison, WI, USA) following manufacturer's instructions.

#### Multi-locus PCR-RFLP genotyping of *T. gondii*

Genotypic characterization of the 24 *T. gondii* isolates was performed using PCR-RFLP analysis of the following 11 DNA segments [8]: SAG1, SAG2 (3′SAG2 and 5′SAG2), SAG2-alt SAG3, BTUB, GRA6, c22-8, c29-2, L358, PK1, and Apico. PCR conditions were the same as previously described [7]. A negative control without DNA was included in each reaction. The RH88 (type I), ME49 (type II) and VEG (type III) strains were used as controls and references. The amplified products were digested using restriction endonucleases (New England BioLabs) specific for each marker [8] according to the manufacturer's instructions. The DNA of the digested products was purified by extraction with an equal volume of phenol/chloroform (1∶1), subjected to polyacrylamide gel (5%) electrophoresis, stained with silver nitrate and photographed.

#### Data analysis

The DNA profiles after restriction enzyme digestion were compared with the reference strains in the virtual ToxoDB database (www.toxodb.org) and compared to the most recent *T. gondii* genotyping results of the animals and humans from Brazil [1]. Additionally, genotyping results of the 24 *T. gondii* isolates were combined with our recently reported genotyping results of *T. gondii* obtained from 24 newborns with congenital toxoplasmosis in Minas Gerais [7], forming a composite dataset of 48 isolates. To determine the genetic relationship of all parasite isolates, the composite dataset of multi-locus PCR-RFLP genotyping was analyzed with SplitsTree4 [13], [14]. The results are presented as a reticulated network to describe the complex relationships of these *T. gondii* isolates. A descriptive analysis was performed to verify the association between the *T. gondii* genotype defined in this study and the virulence of the isolates. Therefore, the data of the virulence in mice was retrieved from previously published reports [3], [10].

### Association between the CS3 Marker and *T. gondii* virulence

#### DNA from the *T. gondii* isolates

DNA obtained from the 48 *T. gondii* isolates was used for genotypic characterization of the CS3 marker. As previously described, eight isolates were obtained from dogs, twelve from free-range chickens, and four from the amniotic fluid of pregnant women who seroconverted during pregnancy [3], [10], [11], [12]. In addition, analysis was performed on DNA samples previously obtained in our laboratory from 24 isolates of *T. gondii* from newborns with congenital toxoplasmosis in Minas Gerais [7].

#### PCR-RFLP at the CS3 locus

The target DNA sequence of the CS3 marker was amplified by PCR using internal primers previously described [5]. Because the DNA was extracted from purified tachyzoites, no previous amplification with the external primers was necessary. The amplification conditions were the same used for the multi-locus PCR-RFLP genotyping [7]; however, 2.5 µL template DNA was used, and the annealing temperature of the cycles was 63°C for 30 seconds. The PCR products were double digested with N1aIII and MboI (New England BioLabs) [5]. The digested products were later purified by extraction with an equal volume of phenol/chloroform (1∶1). The DNA banding pattern was revealed by staining 5% polyacrylamide gels with silver nitrate and photographed. The RH88 (type I), ME49 (type II), and VEG (type III) strains were used as reference.

#### Data analysis

To determine the association between the allele types at the CS3 locus and the virulence phenotypes in mice, the data on mouse virulence were retrieved from three previously published reports [3], [7], [10]. In these studies, groups of mice were intraperitoneally inoculated with 10^0^, 10^1^, 10^2^ e 10^3^ tachyzoites of each isolate. Mice mortality was observed daily throughout 30 days. Isolates killing 100% of the infected mice were classified as virulent. Isolates with a 100% lethal dose (LD_100_) greater than 10^3^ tachyzoites were classified as non-virulent (100% of the infected mice survived, regardless of the dose). Isolates with an intermediate pattern between the two extremes were classified as of intermediate virulence.

The Chi square (χ^2^) or Fisher's exact tests were used to verify the associations between the allele type at the CS3 marker for the isolates and the virulence phenotype for mice [5]. The χ^2^ test with correction by Yates and the Fisher's exact test were used to verify the association between the clinical manifestations of congenital toxoplasmosis and the allele type at the CS3 locus. Therefore, a data bank containing the results of the clinical exams, including the ophthalmologic evaluation and cranial radiographs of the newborns was consulted [7]. The results were considered statistically significant when *p*<0.05. Statistical analysis was performed with the GraphPad Prism 5 software.

## Results

### Genotypic characterization of *T. gondii* isolates from animals and humans in Minas Gerais

Complete genotyping was obtained in 100% of the 24 isolates, and the allele types present in the 11 genetic markers are summarized in Table 1. Twelve different genotypes were identified. Four were classified as new genotypes not previously reported, and eight genotypes had been previously identified (Table 1). Two isolates, CH4 and CH5, matched to genotype ToxoDB #6, also known as Type BrI. Six isolates, D3, D4, CH7, CH9, CH10, and CH11 presented genotype ToxoDB #11, also known as Type BrII. Two isolates, D8 and CH12, presented genotype ToxoDB #8, also known as Type BrIII. These genotypes are considered clonal Brazilian types and were previously identified from domestic animals, such as dogs, cats, and chickens at different regions in Brazil [5]. Genotype BrII had been previously identified in a rabbit isolate in Minas Gerais [15]. One isolate, CH6, presented clonal genotype Type III (ToxoDB #2), rarely described in Brazil [1]. Four isolates, D2, D5, SAF, and RAR, presented genotype ToxoDB #108, previously described in a cat from the state of São Paulo [5]. Two isolates, CH2 and CH3, matched to genotype ToxoDB #19, described in cats, cattle, capybara, chickens, and bats from different Brazilian states [1], [5], [16] as well as in a rabbit from the state of Minas Gerais [15]. One isolate, CH1, matched to genotype ToxoDB #163, previously identified in a free-range chicken from Fernando de Noronha, an archipelago in the Atlantic Ocean approximately 354 km off the Brazilian northeastern coast [17]. Two isolates, CH8 and EFP, matched to genotype ToxoDB #206, recently described in human cases of congenital toxoplasmosis in Minas Gerais [7]. Four new genotypes are designated following the scheme of ToxoDB PCR-RFLP genotype numbers. The new types are ToxoDB #226 (D1), #227 (D6), #228 (D7) and #229 (EGS) (Table 1).

**Table 1:** Multilocus PCR-RFLP genotyping of *Toxoplasma gondii* isolates from human and domestic animals in Minas Gerais, Brazil.

PCR-RFLP Genotype^a^	References	SAG1	5′-3′ SAG2	alt. SAG2	SAG3	BTUB	GRA6	c22-8	c29-2	L358	PK1	Apico	Isolates	Mouse virulence^b^
#10 (Type I)	[7]	I	I	I	I	I	I	I	I	I	I	I	RH88	
#1 (Type II)	[7]	II or III	II	II	II	II	II	II	II	II	II	II	ME49	
#2 (Type III)	[7]	II or III	III	III	III	III	III	III	III	III	III	III	VEG	
#6 (Br I)	[5]	I	I	I	III	I	II	u-1	I	I	I	I	CH4	Virulent
													CH5	Virulent
#11 (Br II)	[5]	I	I	II	III	III	III	I	III	I	II	III	D3	Intermediate
													D4	Intermediate
													CH7	Intermediate
													CH9	Intermediate
													CH10	Virulent
													CH11	Intermediate
#8 (Br III)	[5]	I	III	III	III	III	III	II	III	III	III	III	D8	Avirulent
													CH12	Avirulent
#2 (Type III)	[3]	II or III	III	III	III	III	III	III	III	III	III	III	CH6	Intermediate
#19	[5]	I	III	III	III	III	III	I	I	I	u-1	I	CH2	Intermediate
													CH3	Intermediate
#108	[5]	I	I	II	III	III	III	II	I	I	III	I	D2	Intermediate
													D5	Virulent
													SAF	Virulent
													RAR	Virulent
#163	[17]	I	III	III	III	III	III	II	I	III	III	III	CH1	Intermediate
#206	[7]	u-1	I	II	III	III	III	II	III	I	III	I	CH8	Intermediate
													EFP	Intermediate
#226 (New)	This study	I	I	u-1	III	III	III	II	I	III	III	I	D1	Intermediate
#227 (New)	This study	I	I	II	III	III	III	u-1	I	III	III	I	D6	Virulent
#228 (New)	This study	I	III	III	III	III	III	u-1	I	I	III	I	D7	Intermediate
#229 (New)	This study	I	I	II	III	III	II	I	I	I	II	I	EGS	Virulent

u-1 is a new allele that is different from the clonal type I, II and III alleles.

a According to ToxoDB PCR-RFLP Genotype Number.

b Based on previous studies [3], [10].

**Figure 1:**
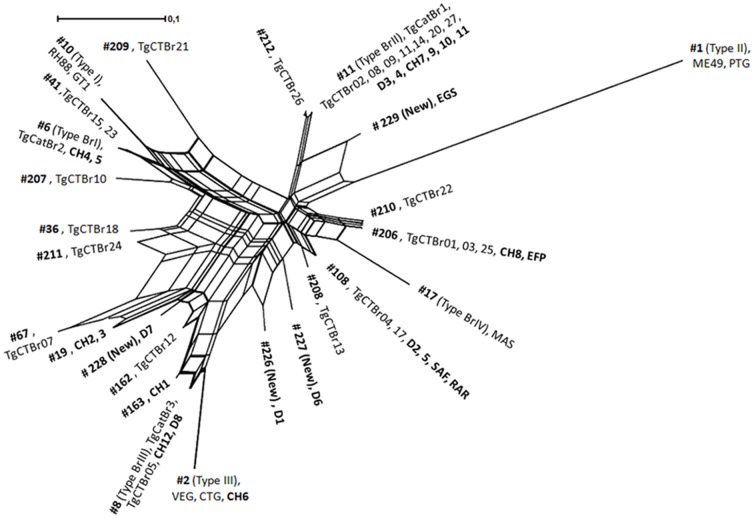
NeighborNet phylogenetic network of *Toxoplasma gondii* isolates from animal and humans in Minas Gerais. Isolates genotyped in this study are highlighted in bold. Isolates TgCTBr01 to TgCTBr26 were obtained from newborns in Minas Gerais and previously genotyped [7].

The genetic relationship of the 24 *T. gondii* isolates from animals and pregnant women genotyped in this study, together with the previously published data from isolates obtained from newborns in Minas Gerais [7] were compared using SplitsTree4 software (Figure 1). The 12 genotypes identified in this study showed high diversity and are scattered in the network. A high degree of similarity was observed between *T. gondii* genotypes circulating in humans and animals, such as dogs and chickens. The most common genotypes among these species in Minas Gerais were ToxoDB #8 (BrIII), ToxoDB #11(BrII), ToxoDB #108, and ToxoDB #206.

The genetic relationship of the 24 *T. gondii* isolates genotyped in this study together with the previously published *T. gondii* genotyping data from domestic animals in other Brazilian regions [1], [5] were also compared. The results confirm the high genotypic diversity of isolates from animal infections in Brazil (data not shown). Genotypes common in Minas Gerais and other Brazilian states were Clonal Type III, BrI, BrII, BrIII, ToxoDB #19, ToxoDB #108, and ToxoDB #163.

Regarding virulence in mice, a descriptive analysis shows an association between the genotype and mouse virulence. Both isolates (100%) with genotype BrI were virulent for mice. Five of the six isolates (83%) with genotype BrII showed intermediate virulence. Two isolates (100%) with genotype BrIII were classified as avirulent (Table 1). The virulence of the CH12 isolate, which is non-virulent for mice, was not determined in the same manner that the virulence of other 11 chicken isolates was determined [10]. However, the two mice used in the bioassay for the primary isolation of the CH12 strain survived up to 30 days after inoculation, indicating low virulence. The DNA was extracted from the tachyzoites after their first passage in mice. This isolate was lost after the second passage because the mice did not become infected (unpublished data). Three of the four isolates (75%) with genotype Toxo DB #108 were virulent. All isolates from genotypes ToxoDB #19 and ToxoDB #206 showed intermediate virulence for mice (Table 1).

### Association between the CS3 marker and *T. gondii* virulence

A total of 48 *T. gondii* isolates were evaluated by PCR-RFLP for the CS3 locus. Twenty-four (50%) presented with allele type I at the CS3 locus, 27% (13/48) presented with allele type II, and 19% (9/48) presented with allele type III. One isolate (2%) presented allele u-1, and one isolate (2%) presented allele u-2 (Table 2). Among the isolates presenting allele type I, 58% presented a virulent phenotype for mice, and 42% presented intermediate virulence (Table 3). All isolates presenting allele type II were either virulent (31%) or of intermediate virulence (69%). No avirulent isolate was found harboring either allele type I or II. Of the isolates presenting allele type III, 44% showed avirulent phenotype, 56% showed an intermediate virulence phenotype and none were virulent. Four avirulent isolates (100%) presented allele type III. Virulence in mice was compared between the three alleles I, II, and III of the CS3 marker (Table 3). Because of the small sample size for the u-1 and u-2 alleles, there was not enough information for the statistical test. Statistical analysis showed a significant difference between alleles I and III (*p* = 0.0001), and between alleles II and III (*p* = 0.0129). No significant difference was observed between alleles I and II (*p* = 0. 1093).

**Table 2:** Distribution of 48 *Toxoplasma gondii* isolates according to allele type at the CS3 locus.

Allele type at the CS3 locus^a^	Number of isolates (%)	*T. gondii* Isolates
I	24 (50%)	D2, D3, D4, D5, D6, CH4, CH5, CH7,
		CH9, CH10, CH11, SAF, EGS, RAR,
		TgCTBr04, TgCTBr08, TgCTBr09,
		TgCTBr11, TgCTBr14, TgCTBr15,
		TgCTBr17, TgCTBr23, TgCTBr26
		and TgCTBr27
II	13 (27%)	CH2, CH3, CH8, EFP, TgCTBr01,
		TgCTBr02, TgCTBr03, TgCTBr07,
		TgCTBr12, TgCTBr13, TgCTBr18,
		TgCTBr22 and TgCTBr25
III	9 (19%)	D1, D7, D8, CH1, CH6, CH12,
		TgCTBr05, TgCTBr10 and TgCTBr16
u-1	1 (2%)	TgCTBr24
u-2	1 (2%)	TgCTBr21
Total	48 (100%)	

a Allele type identified by PCR-RFLP at the CS3 locus in this study.

**Table 3:** Association between mouse virulence and the allele type at the CS3 locus of *Toxoplasma gondii* isolates obtained from animals and humans in Minas Gerais.

Virulence of *T. gondii* a	Allele type at the CS3 locus			
	I	II	III	Total
Avirulent	0	0	4	4
Intermediate	10	9	5	24
Virulent	14	4	0	18
Total	24	13	9	46

a Virulence of *T. gondii* isolate for mice based on previous studies [3], [7], [10].

Regarding the clinical manifestations of congenital toxoplasmosis, of the 24 isolates analyzed by PCR-RFLP at the CS3 locus, 19 originated from newborns with retinochoroiditis. Only three isolates originated from newborns with intracranial calcifications (IC) [7]. The isolate that presented allele u-1 at the CS3 locus (TgCTBr24) was obtained from newborn with retinochoroiditis and the isolate that presented allele u-2 (TgCTBr21) was obtained from newborn with retinochoroiditis and IC. Because of the small sample size of isolates that presented u-1 and u-2 alleles, they were removed from the statistical analysis. No association was found between the allele types at the CS3 locus in *T. gondii* isolates obtained from newborns with congenital toxoplasmosis and clinical signs, either for retinochoroiditis or cerebral calcifications (p>0,05) (Table 4 and Table 5, respectively).

**Table 4:** Association between the allele type at the CS3 locus of *Toxoplasma gondii* isolate and retinochoroidal lesions in newborns with congenital toxoplasmosis (p>0,05).

Allele type at the CS3 locus^a^	Retinochoroidal lesions^b^		
	Present	Absent	Total
I	9	1	10
II	6	3	9
III	2	1	3
Total	17	5	22

a Allele type identified by PCR-RFLP in this study.

b Retinochoroidal lesions described in a previous study [7].

**Table 5:** Association between the allele type at the CS3 locus of *Toxoplasma gondii* isolate and intracranial calcifications in newborns with congenital toxoplasmosis (p>0,05).

Allele type at the CS3 locus^a^	Intracranial calcifications^b^		
	Present	Absent	Total
I	1	9	10
II	1	8	9
III	0	3	3
Total	2	20	22

a Allele type identified by PCR-RFLP in this study.

b Intracranial calcifications described in a previous study [7].

## Discussion

The majority of *T. gondii* isolates genotyped in Brazil were obtained from domestic animals, including free-range chickens, cats, dogs, sheep, and goats [1]. These studies are important because they help identify the population structure of the parasite and provide insight to clarify its epidemiology. However, there are states in Brazil where little is known regarding the genotypic profile of *T. gondii* [1]. Minas Gerais is the second most populous state in Brazil and is located in the southeastern region, where several studies of *T. gondii* have been performed. However, these studies present a gap because the genotyping studies of isolates from animals using 11 previously proposed markers [8] remain rare in this state, rendering it difficult to make correlations with studies performed in other Brazilian regions. The only report of genotyping with these 11 markers performed on *T. gondii* from animals in Minas Gerais used two isolates obtained from rabbits [15]. A genotyping study of *T. gondii* from newborns in this state was recently published [7]. Despite this publication, genotyping studies on human isolates remain rare in Brazil because of the difficulty in obtaining samples [6], [7].

In this study, we identified *T. gondii* genotypes from animals and humans in Minas Gerais and compared them with others previously identified in humans [7] and animals [1], [5] in Brazil. Among the 12 genotypes identified, eight were previously reported at different Brazilian locations. These genotypes include the Brazilian clonal genotypes BrI, BrII, and BrIII, in addition to the clonal genotype Type III. Four genotypes were described for the first time, all presenting only one isolate. This result confirmed the high diversity of *T. gondii* strains in Brazil [1], [5]. However, when the identified genotypes were compared with the *T. gondii* genotypes previously obtained in Minas Gerais [7], an overlapping of the genotypes present in humans and animals was verified. The genotypes commonly circulating among dogs, free-range chickens and humans in Minas Gerais are ToxoDB #8 (BrIII), ToxoDB #11(BrII), ToxoDB #108, and ToxoDB #206. The majority of isolates genotyped in this study were grouped into these profiles. The occurrence of *T. gondii* genotype groups from different host species suggests that there is no host preference for the parasite genotypes. Many genotypes can infect different hosts [1]. Despite the large number of genotyping studies conducted in Brazil, genotype ToxoDB #206 has only been reported in newborns from Minas Gerais [7]. In this study, ToxoDB #206 was identified in two of 24 isolates, one from a free-range chicken and the other from the amniotic fluid of a pregnant woman. These results suggest that isolates from genotypes ToxoDB #8, ToxoDB #11, ToxoDB #108, and ToxoDB #206 have expanded in the state.

It is important to identify the sources of *T. gondii* transmission to humans to understand the epidemiology of toxoplasmosis. Dogs and chickens play an indirect role in the transmission chain of *T. gondii*. Dogs can transmit *T. gondii* mechanically through direct contact with humans, particularly children, because they can harbor sporulated oocysts in their hair [18]. By contrast, direct transmission by ingestion of cysts found in raw or undercooked dog meat is unlikely because these animals are not a source of food for humans in Brazil [19]. *T. gondii* transmission can occur through manipulation of raw free-range chicken meat containing cysts because of a lack of adequate hygiene [20]. The risk of direct transmission through the ingestion of raw or undercooked meat of these animals is low because meat demands a longer time and higher temperature to cook, which is sufficient to kill bradyzoites. Therefore, the genotypic similarity identified in this study suggests the presence of a common source of infection between animals and humans. Oocysts eliminated through cat feces contaminate the environment and are most likely ingested together with water or raw food, representing the primary source of infection [21]. The infection rate of dogs and chickens in Brazil is known to be high, particularly in Minas Gerais [1], [10]. Furthermore, infection in free-range chickens is considered a good indicator of environmental contamination by *T. gondii* oocysts because these animals are raised free and continuously feed on peridomicile soil [10], [20]. Infection in dogs can indicate that parasites and humans have circulated in the same environment because these animals usually live near humans [19]. Other studies have emphasized the importance of transmission by oocysts in Brazil. The risks of uninfected women acquiring toxoplasmosis during pregnancy and fetal transmission are high because of environmental contamination with oocysts [1]. Genotypes BrIII, BrII, and ToxoDB #108 found in this study and in newborns from Minas Gerais [7] were previously identified in cats in other Brazilian regions, including southeastern Brazil [8]. However, further studies on the genotypes circulating in cats in this state are necessary to confirm the occurrence of transmission by oocyst ingestion. Although several studies have emphasized the importance of *T. gondii* transmission by oocysts in Brazil [1], it can not be excluded the possibility of other species of animals being infected with the same genotypes identified in this study, serving as a source of infection by human ingestion of tissue cysts. Therefore, further studies on the genotypes of other animals, such as pigs and sheep, are necessary in Minas Gerais to confirm this possibility.

For virulence in mice, isolates with identical genotypes presented similar phenotypes. These results are consistent with studies that defined the BrI genotype as virulent, BrIII as avirulent, and BrII as intermediate virulent [5], [22]. However, other studies did not find this association [7], [23]. Moreover, the high genetic diversity of *T. gondii* in Brazil [1] makes this association even more complex. Biological differences between the isolates of the same genotype must not be overlooked, and the genetic markers used for genotyping may not appropriately reflect possible phenotypic differences between the isolates [24], [25]. Therefore, it is necessary to study markers that determine *T. gondii* virulence in a more reliable way.

Genetic mapping and positional cloning identified the candidate virulence gene, *ROP18*, encoding a highly polymorphic serine-threonine kinase that was secreted into the host cell during parasite invasion [26]. *ROP18* is located in close proximity to CS3 on chromosome VIIa and it was identified to be the virulence gene of Type I lineage of *T. gondii* [26], [27]. The CS3 marker is probably linked the ROP18 locus, which is expressed in type I and type II strains but not in type III strains. It is likely cause of increased mouse virulence in isolates harboring alleles type I or II at the CS3 locus. Therefore, it is likely that ROP18 is involved in the virulence of those isolates collected from Brazil [5].

The reliability of the CS3 marker in predicting *T. gondii* virulence in the murine model has not been well defined because this relation is not always established [28]. In this study, the analysis of 48 animal and human toxoplasmosis isolates has shown that parasites harboring allele III at the CS3 locus present low virulence for mice, whereas those harboring alleles I or II present higher virulence. These results are consistent with earlier studies showing that alleles I and II are associated with the mortality of infected mice and that allele III is associated with survival [5], [22], [29], [30]. Interestingly, five isolates, D1, D7, CH1, CH6, and TgCTBr16, of intermediate virulence harboring allele III at the CS3 locus were identified. Based on the criteria used to define *T. gondii* virulence [10], the strains with intermediate virulence present a wide range of mortality, from strain causing death at relatively small doses (LD_100_>10 tachyzoites) to strains causing mortality only at high doses (up to 10^3^ tachyzoites). Analysis of the survival time and mortality rates of these five isolates [7], [10] showed that the infected mice presented a longer survival time compared to other isolates with intermediate virulence. In addition, the mortality rates observed at different *T. gondii* dosages were also lower. In most cases, a mortality rate greater than 30% was only observed with large amounts inocula, and even the highest tachyzoite dosage was not sufficient to kill 100% of the animals. Moreover, during isolation of TgCTBr16 strain, the two mice inoculated with newborn blood survived up to 30 days after infection and were only identified as infected by ELISA [7].

In a previous study on the CS3 marker [5], virulence of the *T. gondii* isolates was defined during isolation when mice were inoculated with 1 ml animal tissue preparation containing an unknown number of parasites. Virulent strains presented 100% mortality of the infected mice. Intermediate virulent strains presented mortality greater than 30% but less than 100%, and non-virulent strains presented mortality less than 30% [5]. By contrast, virulence of the isolates genotyped in this study was defined after isolation and was based on the percentage of mortality of BALB/c mice positively infected with graded parasite inocula (10^0^, 10^1^, 10^2^, and 10^3^ tachyzoites) [7], [10]. The isolates were considered avirulent when 100% of the infected mice survived, regardless of the dose. Therefore, differences in the methodology used to define *T. gondii* virulence may explain the results of the strains with intermediate virulence harboring allele III. Several factors interfere with the virulence of *T. gondii*, including parasite stage, infection route, infecting dose, inoculation mode, mouse lineage and intrinsic characteristics of the isolate [31]. Taking these factors into account, the definition of virulence based on known doses of the parasite is more reliable. It had been previously reported that ROP5 and ROP16 (polymorphic pseudokinases and kinase, respectively) are important in *T. gondii* virulence [32], [33]. GRA15 (polymorphic dense granule protein) can modulate host signaling pathways and therefore join rhoptry proteins in *T. gondii*'s host cell–modifying arsenal [34]. So, it is possible that other factors may be influencing the intermediate virulence of the isolates harboring allele III at the CS3 locus, such as these proteins which *T. gondii* secretes into the host cell upon invasion.

This is the first study on the genotyping of a large number of *T. gondii* isolates obtained from domestic animals in Minas Gerais using the 11 markers previously proposed [8]. In addition, it is the first study that verifies the phylogenetic correlation of the genotypes circulating between humans and animals from the same geographic region in Brazil. This study confirms that the CS3 marker is useful for predicting the virulence of Brazilian *T. gondii* isolates in mice and is a more practical and rapid alternative than the murine bioassay. The genotyping results will provide a useful profile for added insight into the population structure, epidemiology and biological characteristics of *T. gondii* [25] in southeastern Brazil.

## References

[1] Dubey JP, Lago EG, Gennari SM, Su C, Jones JL (2012) Toxoplasmosis in humans and animals in Brazil: High prevalence, high burden of disease, and epidemiology. Parasitology 10: 1–50. 10.1017/S0031182012000765 22776427

[2] Howe DK, Sibley LD (1995) *Toxoplasma gondii* comprises three clonal lineages, correlation of parasite genotype with human disease. J Infect Dis 172: 1561–1566. 7594717 10.1093/infdis/172.6.1561

[3] Ferreira AM, Vitor RWA, Gazzinelli RT, Melo MN (2006) Genetic analysis of natural recombinant Brazilian *Toxoplasma gondii* strains by multilocus PCR-RFLP. Infect Genet Evol 6: 22–31. 16376837 10.1016/j.meegid.2004.12.004

[4] Khan A, Jordan C, Muccioli C, Vallochi AL, Rizzo LV, et al (2006) Genetic Divergence of *Toxoplasma gondii* Strains Associated with Ocular Toxoplasmosis, Brazil. Emer Infec Dis 12: 942–949. 10.3201/eid1206.060025 PMC3373049 16707050

[5] Pena HFJ, Gennari SM, Dubey JP, Su C (2008) Population structure and mouse-virulence of *Toxoplasma gondii* . Int J Parasitol 38: 561–569. 17963770 10.1016/j.ijpara.2007.09.004

[6] Ferreira IMR, Vidal JE, De Mattos CCB, De Mattos LC, Qu D, et al (2011) *Toxoplasma gondii* isolates: Multilocus RFLP PCR genotyping from human patients in Sao Paulo State, Brazil identified distinct genotypes. Exp Parasitol 129: 190–195. 21741380 10.1016/j.exppara.2011.06.002

[7] Carneiro AC, Andrade GM, Costa JG, Pinheiro BV, Vasconcelos-Santos DV, et al (2013) Genetic characterization of *Toxoplasma gondii* revealed highly diverse genotypes for isolates from newborns with congenital toxoplasmosis in Southeastern Brazil. J Clin Microbiol 51: 901–907. 23284022 10.1128/JCM.02502-12 PMC3592078

[8] Su C, Shwab EK, Zhou P, Zhu X, Dubey JP (2010) Moving towards and integrated approach to molecular detection and identification of *Toxoplasma gondii* . Parasitology 137: 1–11. 19765337 10.1017/S0031182009991065

[9] Khan A, Taylor S, Su C, Mackey AJ, Boyle J, et al (2005) Composite genome map and recombination parameters derived from three archetypal lineages of *Toxoplasma gondii* . Nuc Acids Res 33: 2980–2992. 10.1093/nar/gki604 PMC1137028 15911631

[10] Brandão GP, Ferreira AM, Melo MN, Vitor RWA (2006) Characterization of *Toxoplasma gondii* from domestic animals from Minas Gerais. Parasite 13: 143–149. 16800123 10.1051/parasite/2006132143

[11] Vidigal PV, Santos DV, Castro FC, Couto JC, Vitor RWA, et al (2002) Pre-natal toxoplasmosis diagnosis from amniotic fluid by PCR. Rev Soc Bras Med Trop 35: 1–6. 10.1590/s0037-86822002000100001 11873253

[12] Castro FC, Viegas Castro MJB, Cabral ACV, Brasileiro Filho G, Vitor RWA, et al (2001) Comparação dos Métodos para Diagnóstico da Toxoplasmose Congênita. Rev Bras Ginecol Obstet 23: 277–282.

[13] Huson D (1998) SplitsTree: analyzing and visualizing evolutionary data. Bioinformatics 14: 68–73. 9520503 10.1093/bioinformatics/14.1.68

[14] Huson DH, Bryant D (2006) Application of phylogenetic networks in evolutionary studies. Mol Biol Evol 23: 254–267. 16221896 10.1093/molbev/msj030

[15] Dubey JP, Passos LMF, Rajendran C, Ferreira LR, Gennari SM, et al (2011) Isolation of viable *Toxoplasma gondii* from guinea fowl (*Numida meleagris*) and domestic rabbits (*Oryctolagus cuniculus*) from Brazil. J Parasitol 97: 842–845. 21506805 10.1645/GE-2728.1

[16] Cabral AD, Gama AR, Sodré MM, Savani ESMM, Galvão-Dias MA, et al (2013) First isolation and genotyping of *Toxoplasma gondii* from bats (Mammalia: Chiroptera). Vet Parasitol 193: 100–104. 23200751 10.1016/j.vetpar.2012.11.015

[17] Dubey JP, Rajendran C, Costa DG, Ferreira LR, Kwok OC, et al (2010) New *Toxoplasma gondii* genotypes isolated from free-range chickens from the Fernando de Noronha, Brazil: unexpected findings. J Parasito. 96: 709–712. 10.1645/GE-2425.1 20486738

[18] Lindsay DS, Dubey JP, Butler JM, Blagburn BL (1997) Mechanical transmission of *Toxoplasma gondii* oocysts by dogs. Vet Parasitol 73: 27–33. 9477489 10.1016/s0304-4017(97)00048-4

[19] Germano PML, Erbolato EB, Ishizuka MM (1985) Estudo sorológico da toxoplasmose canina pela prova da imunofluorescência indireta na cidade de Campinas. Rev Fac Med Vet Zootec USP 22: 53–58.

[20] Literák I, Hejlícek K (1993) Incidence of *Toxoplasma gondii* in populations of domestic birds in the Czech Republic. Avian Pathol 22: 275–281. 18671017 10.1080/03079459308418920

[21] Dubey JP, Lindsay DS, Speer C (1998) Structures of *Toxoplasma gondii* tachyzoites, bradyzoites and sporozoites and biology and development of tissue cysts. Clin Microbiol Rev 11: 267–299. 9564564 10.1128/cmr.11.2.267 PMC106833

[22] Yai LE, Ragozo AM, Soares RM, Pena HF, Su C, et al (2009) Genetic diversity among capybara (*Hydrochaeris hydrochaeris*) isolates of *Toxoplasma gondii* from Brasil. Vet Parasitol 162: 332–337. 19375864 10.1016/j.vetpar.2009.03.007

[23] Frazão-Teixeira E, Sundar N, Dubey JP, Grigg ME, Oliveira FCR (2011) Multi-locus DNA sequencing of *Toxoplasma gondii* isolated from Brazilian pigs identifies genetically divergent strains. Vet Parasitol 175: 33–39. 21051148 10.1016/j.vetpar.2010.09.030 PMC3141217

[24] Soares RM, Silveira LH, Silva AV, Ragozo A, Galli S, et al (2011) Genotyping of *Toxoplasma gondii* isolates from free range chickens in the Pantanal area of Brazil. Vet Parasitol 178: 29–34. 21255933 10.1016/j.vetpar.2010.12.037

[25] Wang L, Chen H, Liu D, Huo X, Gao J, et al (2013) Genotypes and mouse virulence of *Toxoplasma gondii* isolates from animals and humans in China. PLoS One 8: e53483. 23308233 10.1371/journal.pone.0053483 PMC3538538

[26] Taylor S, Barragan A, Su C, Fux B, Fentress SJ, et al (2006) A Secreted Serine-Threonine Kinase Determines Virulence in the Eukaryotic Pathogen *Toxoplasma gondii* . Science 314: 1776–1780. 17170305 10.1126/science.1133643

[27] Khan A, Taylor S, Ajioka JW, Rosenthal BM, Sibley LD (2009) Selection at a single locus leads to widespread expansion of *Toxoplasma gondii* lineages that are virulent in mice. PLoS Genet 5: e1000404. 19266027 10.1371/journal.pgen.1000404 PMC2644818

[28] Silva RC, Langoni H, Su C, Silva AV (2011) Genotypic characterization of *Toxoplasma gondii* in sheep from Brazilian slaughterhouses: new atypical genotypes and the clonal type II strain identified. Vet Parasitol 175: 173–177. 20970257 10.1016/j.vetpar.2010.09.021

[29] Ragozo AMA, Pena HFJ, Yai LEO, Su C, Gennari SM (2010) Genetic diversity among *Toxoplasma gondii* isolates of small ruminants from Brazil: Novel genotypes revealed. Vet Parasitol 170: 307–312. 20236768 10.1016/j.vetpar.2010.02.024

[30] Pena HFJ, Vitaliano SN, Beltrame MAV, Pereira FEL, Gennari SM, et al (2013) PCR-RFLP genotyping of *Toxoplasma gondii* from chickens from Espírito Santo state, Southeastern region, Brazil: New genotypes and a new SAG3 marker allele. Vet Parasitol 192: 111–117. 23116899 10.1016/j.vetpar.2012.10.004

[31] Dubey JP, Graham DH, Blackston CR, Lehmann T, Gennari SM, et al (2002) Biological and genetic characterization of *Toxoplasma gondii* isolates from chickens (*Gallus domesticus*) from São Paulo Brazil: unexpected findings. Int J Parasitol 32: 99–105. 11796127 10.1016/s0020-7519(01)00364-2

[32] Reese ML, Zeiner GM, Saeij JP, Boothroyd JC, Boyle JP (2011) Polymorphic family of injected pseudokinases is paramount in *Toxoplasma* virulence. Proc Natl Acad Sci USA 108: 9625–9630. 21436047 10.1073/pnas.1015980108 PMC3111280

[33] Saeij JP, Boyle JP, Coller S, Taylor S, Sibley LD, et al (2006) Polymorphic secreted kinases are key virulence factors in toxoplasmosis. Science 314: 1780–1783. 17170306 10.1126/science.1133690 PMC2646183

[34] Rosowski EE, Lu D, Julien L, Rodda L, Gaiser RA, et al (2011) Strain-specific activation of the NF-kappaB pathway by GRA15, novel *Toxoplasma gondii* dense granule protein. J Exp Med 208: 195–212. 21199955 10.1084/jem.20100717 PMC3023140

